# WNT Signaling in Stem Cells: A Look into the Non-Canonical Pathway

**DOI:** 10.1007/s12015-023-10610-5

**Published:** 2023-10-07

**Authors:** Miguel Angel Sarabia-Sánchez, Martha Robles-Flores

**Affiliations:** Departamento de Bioquímica, Facultad de Medicina, Universidad Nacional Autónoma de México (UNAM), Mexico City, Mexico

**Keywords:** Non-canonical Wnt, Stemness, Cancer, Cancer stem cell

## Abstract

Tissue homeostasis is crucial for multicellular organisms, wherein the loss of cells is compensated by generating new cells with the capacity for proliferation and differentiation. At the origin of these populations are the stem cells, which have the potential to give rise to cells with both capabilities, and persevere for a long time through the self-renewal and quiescence. Since the discovery of stem cells, an enormous effort has been focused on learning about their functions and the molecular regulation behind them. Wnt signaling is widely recognized as essential for normal and cancer stem cell. Moreover, β-catenin-dependent Wnt pathway, referred to as canonical, has gained attention, while β-catenin-independent Wnt pathways, known as non-canonical, have remained conspicuously less explored. However, recent evidence about non-canonical Wnt pathways in stem cells begins to lay the foundations of a conceivably vast field, and on which we aim to explain this in the present review. In this regard, we addressed the different aspects in which non-canonical Wnt pathways impact the properties of stem cells, both under normal conditions and also under disease, specifically in cancer.

**Graphical abstract:**

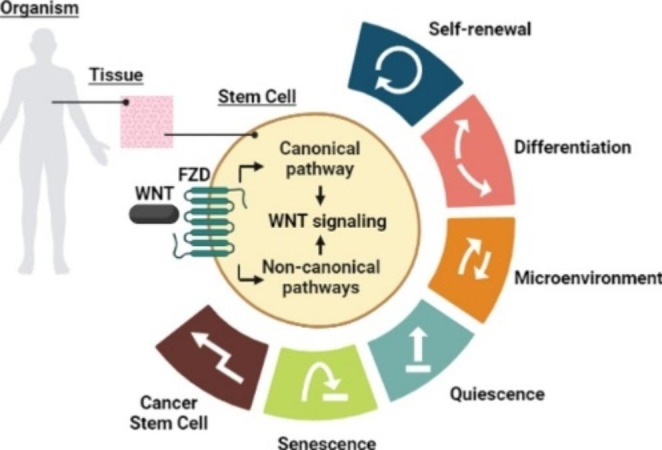

## Introduction

The canonical Wnt pathway has been extensively supported to be critical in stem cell biology and functions, but little is known about the roles of non-canonical Wnt pathways in these cells [1]. Stem cells are crucial for physiological tissue renewal and regeneration due to their ability to self-renew and to give rise to multiple cell types by differentiation [2]. The maintenance of the undifferentiated state of stem cells in adulthood is achieved due to self-renewal, wherein the biological characteristics and developmental potential are preserved despite going through cell division [3]. Self-renewal allows stem cells to persist. However, in addition to self-renewal, quiescence is part of the stem cell strategy to be available during the adult stage because it implies a low cell cycle rate, which consequently establishes two populations of stem cells: quiescent and proliferating/active stem cells [4]. In specific conditions, the stem cells differentiate into progenitors, giving rise to the cell lineages corresponding to the tissue where they reside. However, the stem cells can enter a senescent state wherein the potential to proliferate and differentiate is missing [5]. For this reason, the microenvironment or niche plays a decisive role in keeping stem cells in an optimal state, in which the secretion of Wnt ligands is indispensable [6]. Recent research on stem cells has revealed that the non-canonical Wnt pathways enable stem cells to carry out their tasks.

Noteworthy, the canonical and non-canonical Wnt pathways were initially conceived as linear and independent mechanisms, but nowadays, it has been demonstrated that they share several signaling components, supporting the idea of a whole signaling network. As previously mentioned, the findings reinforce the notion that that one ligand can promote simultaneous activation under specific conditions [7]. However, in this review, we were interested in explaining the findings that discern the regulation of traits of stem cells by non-canonical Wnt pathways as complementary knowledge to what is known for the canonical Wnt pathway, even under disease conditions such as cancer.

## Wnt Pathways

**Fig. 1:**
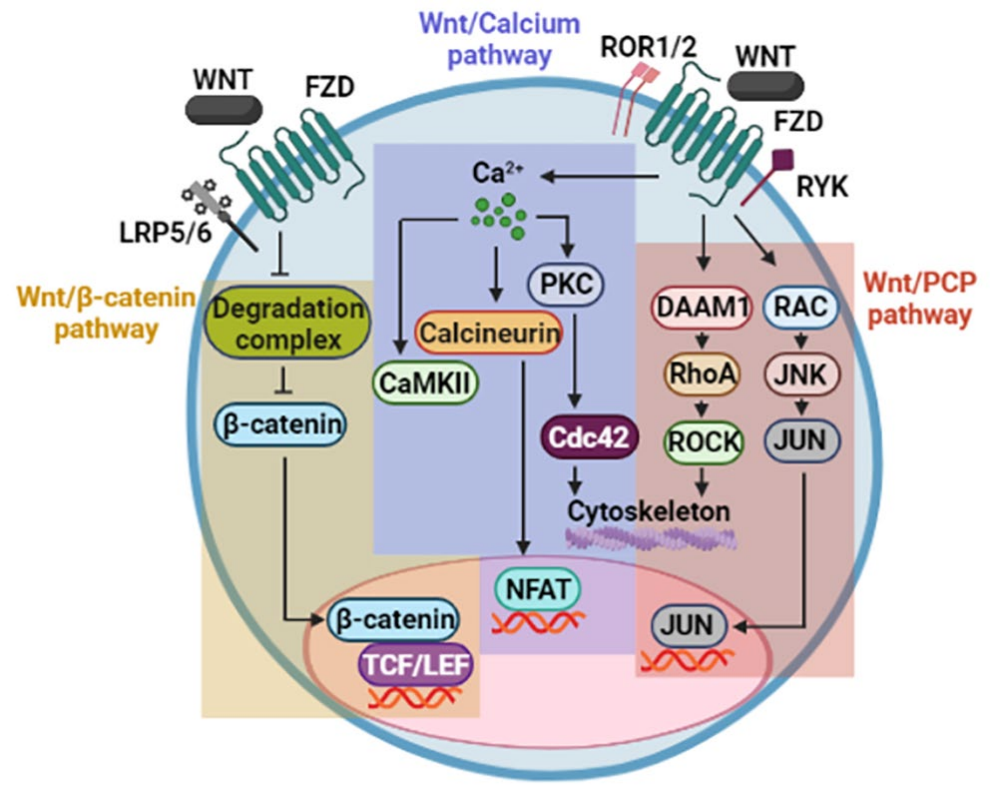
**Wnt signaling network.** The canonical Wnt pathway is mediated by β-catenin-dependent transcription. Typical non-canonical signaling comprises Wnt/calcium and Wnt/PCP pathways, both of which cause activation of members of the GTPase family, with modulation of gene expression and cytoskeletal rearrangements. Wnt/calcium pathway activates effectors such as CaMKII, Calcineurin, and PKC through oscillations in intracellular calcium levels, in addition to upregulating NFAT. Meanwhile, Wnt/PCP pathway stimulates RAC and RhoA, as well as the transcriptional activity of JUN.

The Wnt family consists of cysteine-rich secreted glycoproteins that act as ligands in an autocrine and paracrine manner to regulate a wide range of cellular functions [8]. Within the transduction mechanisms, the β-catenin-dependent signaling has been the first and most widely described, being categorized as the canonical Wnt pathway [9]. However, it is known that Wnt ligands can elicit β-catenin-independent mechanisms, classifying them as non-canonical Wnt pathways. Specifically, Wnt/calcium and PCP (Planar Cell Polarity) pathways have been well-studied non-canonical Wnt pathways [8]. Briefly, the Wnt/calcium pathway leads to increased intracellular calcium levels and, hence, the activation, but not exclusively, of CAMKII, PKC, and Calcineurin. Interestingly, this pathway, in addition to leading to NFAT-mediated transcriptional activation downstream of Calcineurin, also triggers cytoskeletal rearrangement by improving the activity of CDC42, a target of PKC [10]. Meanwhile, the PCP pathway involves the signaling module DAAM/RHOA/ROCK, which has an effect on actin polymerization [11]. Therefore, WNT/calcium and Wnt/PCP pathways influence the cell polarity and migration [12, 13]. In the case of the PCP pathway, the genetic expression profile can be modified through JUN downstream of JNK, and JNK, in turn, is activated by RAC1, a parallel component of the PCP pathway [8] (Fig. 1).

New components in both types of Wnt pathways are being discovered, while some already described are associated with a specific Wnt pathway [14, 15]. For example, Wnt3a and Wnt5a are referred to as prototype ligands that activate canonical and non-canonical Wnt pathways, respectively [16]. As will be seen throughout the present review, these ligands are indicators of whether one or another pathway is activated; however, it is crucial to take into account that the same Wnt ligand may activate both canonical and non-canonical Wnt pathways simultaneously [7].

Recognition of Wnt ligands requires Fzd (Frizzled) receptors, which can be assisted by co-receptors, which serve as accessories and cooperate in determining the specificity of the downstream effectors. Like Wnt3a and Wnt5a, co-receptors are commonly related to one particular Wnt signaling. The LRP5/6 co-receptor employs the canonical Wnt pathway, while ROR1/2 and Ryk are linked to the activation of non-canonical Wnt pathways [16]. ROR1/2 are atypical RTK co-receptors, and Ryk can act as a receptor or co-receptor [17].

## The Perseverance to Keep Walking: Non-Canonical Wnt Pathway as a Path to Self-Renewal

A hallmark characteristic of stem cells is their self-renewal capacity, which allows them to preserve their undifferentiated state features and maintain the potential to specialize towards specific cell lineages [3]. It has been challenging to assess the self-renewal ability; however, there are methods to address it, such as the ability to reconstitute hematopoiesis in the case of in vivo transplantation of HSC (Hematopoietic Stem Cells) or the ability to form spheres, one of the most widely used assays under culture conditions, that is considering as standard test in the study of the self-renewal.

Although the hematopoiesis reconstitution is considered an in vivo gold functional test of HSC, transplantation into serial recipients is also required to demonstrate the long-term clonal growth [18]. The expression of the non-canonical Wnt5a ligand in primitive hematopoietic cells [7, 12–17] has emphasized the essential role of non-canonical Wnt signaling in replenishing the hematopoietic system [19]. Indeed, an assay of hematopoiesis reconstitution of xenotransplanted mice with human HSCs demonstrated that CD34^+^CD38^−^Lin^−^ cells were enriched in Wnt5a-treated mice and that the engrafted mice repopulated more efficiently with Wnt5a-treated HSCs than unstimulated HSCs.

Regarding the canonical Wnt pathway, although the activity of β-catenin in response to Wnt3a has been proven to be important for the self-renewal of HSC [20, 21], the deletion of β-catenin in bone marrow-derived precursors displayed a similar potential for hematopoietic repopulation than wild-type precursors. Because no detection of β-catenin in mature blood cells was described, the dispensable role of β-catenin in the hematopoietic process under physiological conditions was proposed [22, 23]. Consistent with this, it has been reported recently [24] that using Wnt3a and Wnt5a as prototype ligands to activate the canonical or the non-canonical pathways, respectively, in colon cancer stem cells (CSCs), both ligands promote sphere-formation capacity and proliferation in a β-catenin-independent manner. Both Wnt3a and Wnt5a were also found to induce or maintain sphere formation by the downstream activation of Phospholipase C and transcriptional factor NFAT. The single specific inhibition of PLC or NFAT leads to impaired sphere formation, indicating, therefore, that the non-canonical Wnt/Ca2 + signaling activated by both Wnt ligands is essential to induce/maintain the self-renewal efficiency of the CSCs [24].

The relevance of the non-canonical Wnt pathway in Melanocyte precursor cells (MPCs) has been demonstrated for the preservation of a less-differentiated state. MPCs obtained from iPSCs (induced pluripotent stem cells) and comparable to melanocyte stem cells expressed high levels of Wnt5a and non-canonical ROR2 co-receptor. Because the knockdown of ROR2 triggered MPCs differentiation by activating the canonical WNT pathway, it was suggested that non-canonical Wnt partially favors a less-differentiated state, likely involving JUN due to its upregulation in MPCs [3].

In addition, higher levels of Wnt5a, Ror2, and Fzd1 were detected in Mesenchymal Stem Cells (MSCs) derived from bone marrow (BM) and umbilical cord blood than in human iPSCs [25]. In this report, the expression profile of surface proteins in MSC was maintained by circFOXP1, a circRNA originating from the *FOXP1* gene. This circFOXP1 acts as a miRNA sponge targeting miR-17-3p and miR-127-5p. Both miRNAs downregulate WNT5A and ROR2 mRNAs. Thereby circFOXP1 sustains non-canonical Wnt in MSC through increasing levels of WNT5A and ROR2 [25].

The canonical Wnt pathway has been demonstrated to be important in the efficiency of forming spheres when, for example, normal neural stem cells (NSC) are exposed to Wnt3a without affecting the range of cell division in the spheres [26]. In mammary epithelial cells (MEC), both Wnt3a and Wnt5a increased the capacity to form mammospheres wherein each type of ligand induced distinct signaling pathways. Wnt3a upregulated the canonical Wnt signaling dependent on Lrp5/6, while Ror2 and JNK activities were necessary for Wnt5a stimulus [27]. Indeed, recently it has been reported that using Wnt3a and Wnt5a as prototype ligands to activate the canonical or the non-canonical pathways, respectively, promotes sphere-formation capacity and proliferation through the β-catenin-independent manner in colon cancer cells [24]. Both Wnt3a and Wnt5a were found to induce or maintain sphere formation by triggering Phospholipase C (PLC) and transcriptional factor NFAT activation. Consistent with this, the inhibition of PLC or NFAT impaired sphere formation, indicating that the non-canonical Wnt/calcium signaling activated by both Wnt ligands is essential for self-renewal of CSC, discussed in detail later in the text [24].

**Fig. 2:**
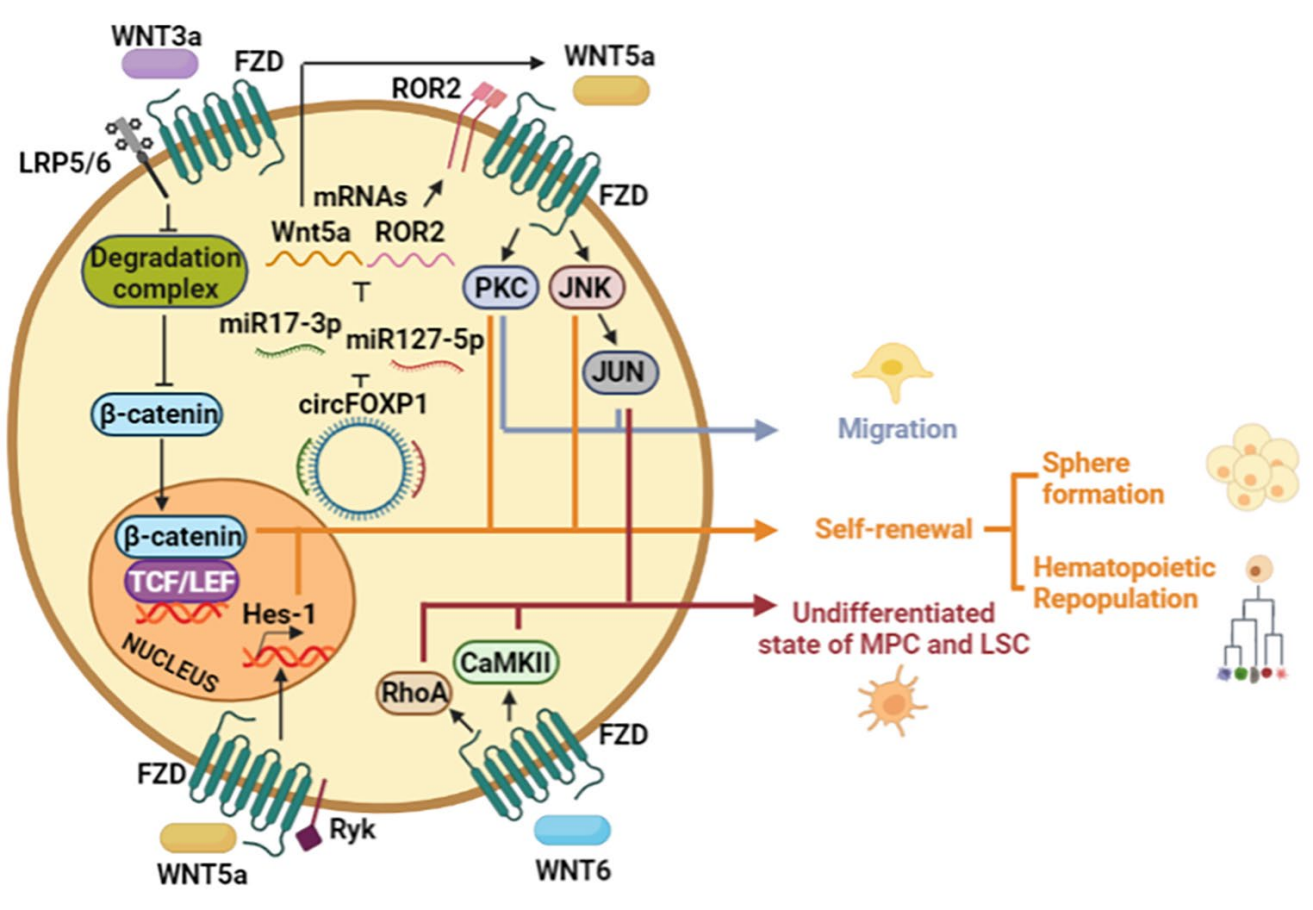
**Regulation of self-renewal by Wnt ligands.** Wnt3a and Wnt5a confer self-renewal capacity, where Wnt3a increases β-catenin activity, while Wnt5a stimulates JNK and PKC. Furthermore, PKC and JUN stimulate the migratory capacity of stem cells. Wnt5a and ROR2 mRNAs are downregulated by miR17-3p and miR-127-5P. However, these miRNAs are inhibited by circFOXP1. Wnt5a can elicit the expression of Hes-1, a component of the Notch pathway, to allow the self-renewal of HSC. In the case of MPC and LSC, the undifferentiated state is maintained by JUN, RhoA, and CaMKII.

Exploration of the self-renewal has revealed that Wnt5a and components of the non-canonical Wnt pathway act as molecular intermediaries cooperating in maintaining the undifferentiated state [28]. Therefore, non-canonical Wnt ligands might be secreted to prevent premature differentiation and maintaining self-renewal simultaneously [29]. However, as discussed below, this seems to depend on the cellular context, as non-canonical Wnt signaling promotes differentiation in specific circumstances [30, 31]. Since self-renewal has been associated with other capabilities, such as cell migration [32] and hematopoietic repopulation [23, 29], it would be interesting to know what other cellular functions are closely related to self-renewal capacity, which are regulated by the non-canonical Wnt pathway (Fig. 2).

## Acquiring a New Destiny: Non-canonical Wnt Pathway as a Passport to Differentiation

According to the requirements of the tissues, the stem cells begin the differentiation process to compensate for the loss of cells, either through normal attrition or in response to pathological conditions [43]. Although it was previously mentioned that Wnt5a could sustain the undifferentiated state of stem cells, Wnt5a participates in the differentiation of other cell types, which emphasizes that the role of Wnt5a in differentiation depends on the traits of the biological model under study [44].

The differentiation capacity towards one or several cell lineages depends on the degree of the stem cell potentiality. In the case of multipotent cells, the balance in the expression between two prototypical canonical and non-canonical ligands, Wnt3a and Wnt5a, respectively, can dictate the specific lineage acquired. For instance, Wnt3a favors lymphopoiesis, while Wnt5a augments myelopoiesis in the BM and spleen; however, it is necessary to know the mechanisms triggered by each one [45].

MSCs categorized as multipotent progenitors have attracted attention because MSCs can be isolated from different anatomical locations and obtain a specific cell type to replace damaged cells when transplanted [33]. Within MSC-derived cell lineages, osteogenic differentiation has provided insights into the role of the non-canonical Wnt pathways in differentiation. Indeed, experimental evidence of the Calcium-dependent signaling participation in osteogenic differentiation has been reported [34]. Previous studies showed that Wnt3a increased nuclear β-catenin levels, but also NKD1 (Naked cuticle 1 homolog), Wnt11, and Wnt5a expression in human MSCs during osteogenesis. Activation of JNK stimulated osteogenic differentiation while repressed adipogenic differentiation, confirming additional mechanisms to those dependent on β-catenin in decision-making towards a specific cell lineage. It is conceivable that the non-canonical Wnt signaling emerges because Wnt5a was upregulated in this phenomenon [35]. Consistently, tendon-derived stem cells can induce osteogenic differentiation upon uniaxial mechanical tension (UMT). During the process, mechanical stress upregulated the levels of Wnt5a, Wnt5b, Ror2, and Rac1. Moreover, the absence of Wnt5a or Wnt5b impaired UMT-induced Runx2 expression, but this repressed effect was reversed when JNK1 was overexpressed, revealing that Wnt5a or Wnt5b promotes osteogenic differentiation through JNK activation [36]. In MSC obtained from human adult oral and craniofacial tissues, Wnt4 is shown to be a positive regulator in osteogenic differentiation without affecting cell proliferation or death. Besides, p38 MAPK augmented while β-catenin levels did not undergo changes in the presence of Wnt4. Even an autocrine mechanism by another stimulus is unlikely due to the short time response of p38 activation [30]. Interestingly, p38 phosphorylation induced by Wnt4 was dependent on Axin, which did not alter its levels, so the interaction of Axin with MEKK1, an activator of JNK, could be a mode of action [37].

The notion of Wnt mediating calcium oscillations in osteogenic differentiation has also been strengthened by other findings in adipocyte-derived stem cells (ADSCs), in which the overexpression of miR-26a-5p, a miRNA targeting mRNA of Wnt5a, reduced the intracellular calcium concentration, protein levels of CaMKII and phosphorylated form of PKC in these cells [38]. Besides, high levels of miR-26a-5p impaired osteogenic differentiation while levels of β-catenin were increased, indicating that the canonical Wnt pathway must be repressed to allow the cellular process [53]. The inhibitor effect of the canonical Wnt pathway could explain why the Wnt ligands restrict osteogenic differentiation in some cases, demonstrating that Wnt ligands can elicit opposite responses depending on the downstream effectors [39, 40].

During an early stage of differentiation, stem cells may divide symmetrically or asymmetrically. In asymmetric division, one daughter cell remains a stem cell, and the other is a non-stem cell [41]. In osteogenic induction of MSCs, they proliferate asymmetrically, and Osterix and OCT4 are expressed in differentiated cells and undifferentiated cells, respectively [42]. Sr (Strontium), an osteogenic compound, increases the proportion of Osterix^+^ cells, but the ratio of Osterix^−^ cells remains similar, revealing that the MSC pool is maintained while generating differentiated cells [42]. During Sr treatment, both Wnt3a and Wnt5a were augmented, and the use of Box-5, a Wnt5a antagonist, reversed Sr-mediated downregulation of aPKC and Par3, components of PAR complex. Because the reduction of aPKC and Par3 occurred simultaneously with a diminished asymmetric distribution between Osterix^+^ and Osterix^−^ cells, the role of Wnt5a was associated with proper asymmetric division. Additionally, Ror2 and Fzd4 were proposed to participate in this axis signaling due to their increase upon Sr treatment [42].

Although many findings support the fact that Wnt5a contributes to osteogenic differentiation, exceptions have been described. For example, in human periodontal ligament-derived stem cells, Wnt5a suppressed the osteogenic differentiation, wherein Ror2 was responsible for triggering the phosphorylation of JNK [43]. This evidence highlights that the cell context influences the action of Wnt5a, and therefore, the relationship between Wnt ligands and the differentiation must be analyzed carefully.

MSCs have been reported to be capable of differentiating into cell types other than mesenchymal, such as neuronal lineage, which could be an alternative treatment for neurodegenerative diseases [44]. In another study, Wnt3a increased neuron and astrocyte markers in BM-MSC induced by β-mercaptoethanol, wherein the levels of RhoA, c-Jun, and Wnt3a were augmented, suggesting a role of non-canonical Wnt/PCP (cell polarity) pathway, but further studies are required because the formation of neurons was not examined [45]. Regarding the neuronal differentiation, Wnt3a increased intracellular calcium levels and, as a consequence, stimulated the active form of CaMKII and Pyk2 (Proline-rich tyrosine kinase 2) in human neural progenitor cells. Worthy of mentioning, Pyk2 negatively regulated the active form of GSK3β and favored the stabilization of β-catenin [46]. Whether the effect of Pyk2 on β-catenin is through a direct or indirect mechanism remains to be elucidated because Pyk2 can interact with β-catenin to mediate its phosphorylation status [47]. In contrast, downregulation of the canonical Wnt pathway has been described in the presence of Wnt3a during neurogenic differentiation of human embryonic stem cell-derived neural progenitor cells [48]. Furthermore, JNK and ATF2 were required to promote neural precursor specification, acting through CREB, c-Jun, and AP-1 downstream. In contrast, DKK1 did not affect neuronal gene expression, supporting the notion of canonical Wnt-independent mechanisms, which was similar to primary NSC [48]. Therefore, regulating the canonical Wnt pathway in neurogenic differentiation may depend on the stem cells origin.

In a subpopulation of Intestinal Stem Cells (ISCs), the switch from Wnt/β-catenin pathway to Wnt/PCP pathway activation has been demonstrated, wherein an upregulation of Vangl2, Dvl2, Ror2, and Celsr1 was detected [49]. Wnt/PCP signaling was associated with the lineage priming toward Paneth cells and Enteroendocrine cells through unipotent progenitors, in which JNK activity was implicated, and Fltp (Flattop) found to be the effector [49].

There is considerable evidence establishing the pivotal role of non-canonical Wnt signaling in embryonic development, and we focus on Embryonic Stem Cell (ESC) differentiation [50]. Retinoic acid (RA) reduces stem cell marker Lrh-1 (Liver receptor homolog-1) and increases differentiation markers Cyr61 (Cysteine-rich angiogenic inducer 61) and Zic3 (Zinc finger protein family member 5) in ESC. Likewise, increased expression of canonical and non-canonical Wnt ligands, including Wnt3a and Wnt5a, was observed with RA treatment. Meanwhile, the transcriptional reporter activity of β-catenin was repressed, and NFAT activity was stimulated. Thus, RA seems to improve non-canonical Wnt signaling. Interestingly, Tcf3, a typical partner of β-catenin, was localized in promoters of Lrh-1, Sox2, and Oct4 upon RA treatment; thereby, Tcf3 functions dually, either as a transcriptional activator or repressor, according to the cellular context [51].

In adult lung epithelial progenitors, the β-catenin/p300 complex is an example in which β-catenin acts together with its interaction partner to drive differentiation toward AT1 cell-like phenotype, wherein components of non-canonical Wnt pathway perform as cooperative effectors [31]. Specifically, Wnt5a promoted the formation of p300/ β-catenin complex through the phosphorylation of p300 at Ser-89, for which PKCζ was proposed as the possible responsible kinase [31]. Interaction between p300 and β-catenin resulted in being important for the specialization of non-epithelial cells so that it could be similar in other types of differentiation [31].

**Fig. 3:**
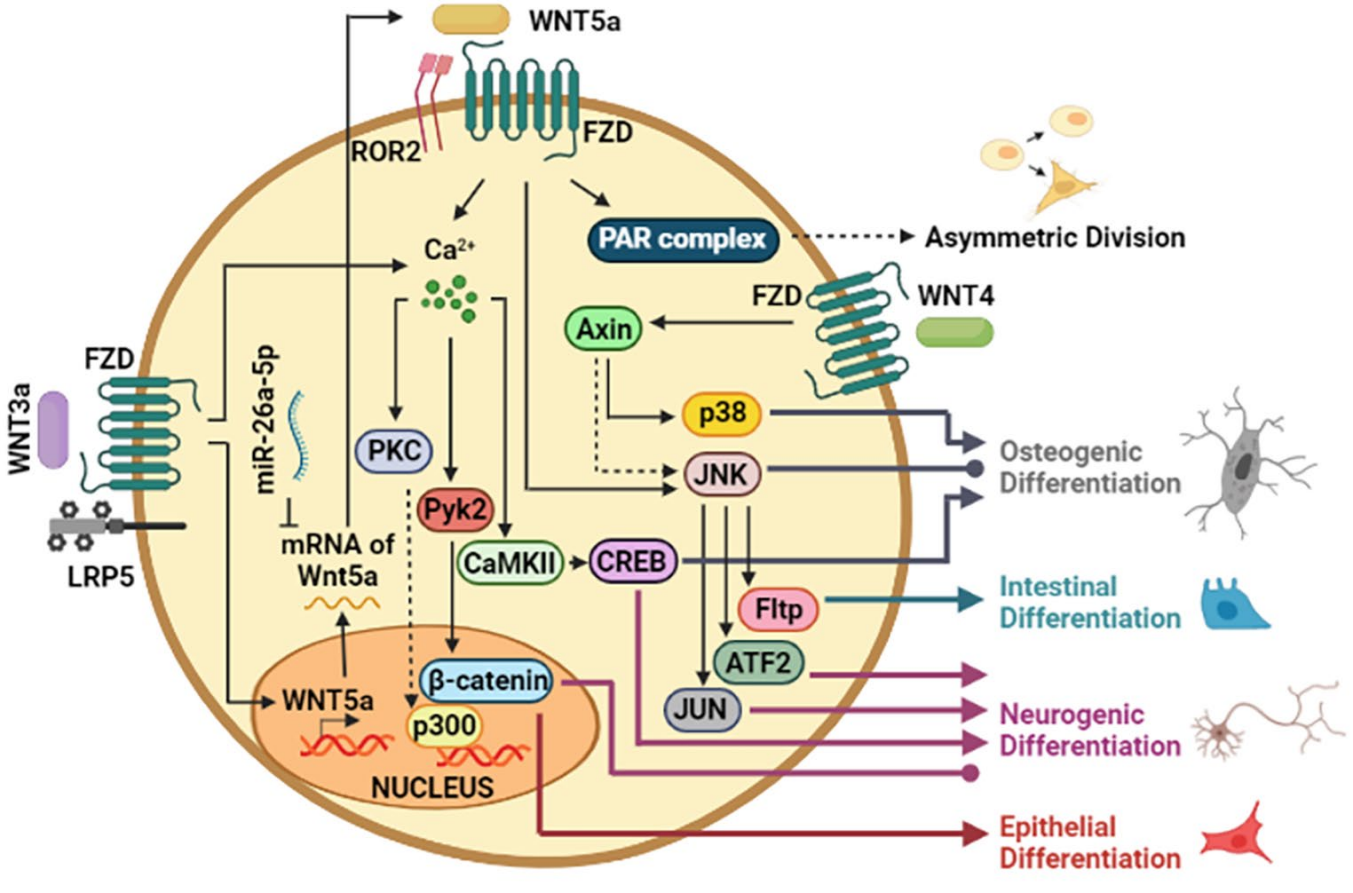
**Differentiation in response to Wnt.** In specific stem cells, Wnt3a induces the expression of Wnt5a, in which Wnt5a mRNA can be targeted by miR-26a-5p. Wnt5a increases intracellular calcium levels and JNK activity to regulate downstream targets, including β-catenin. Possible direct interactions between effectors are indicated (dotted line). Positive regulation of differentiation is indicated (arrowhead line). Nevertheless, effectors described as activators and inhibitors of specific lineage are also indicated (round point line). Asymmetric division, as an initial step in differentiation, is dictated by Wnt5a through the function of the PAR complex. The role of other Wnt ligands, such as Wnt4, in osteogenic differentiation is shown

The study of differentiation has proven to be a complex process since different types of cells can give rise to the same differentiated cell, whether evaluated from molecular markers or functional assays [33]. However, the notion that Wnt signaling contributes to cell specification suggests that other types of differentiation may be dictated by β-catenin-independent mechanisms (Fig. 3).

## We are Not Alone in this Universe: Non-Canonical Wnt Pathway in Communicating with the Normal and Cancer Conditions Microenvironment

The interaction between the microenvironment and stem cells is crucial to perform their functions because the cell identity is partially conferred by extrinsic factors, thus defining a specific niche for stem cells [52]. Progress on this issue has been achieved mainly in intestinal crypts, in which the telocytes, an interstitial cell type, are essential to provide Wnt ligands to the niche of ISC. Moreover, the function of telocytes could be shared due to their presence in various epithelial tissues [53]. Several studies have demonstrated the activation of the non-canonical Wnt pathway in niche-forming cells involved in the maintenance of stem cells [54, 55]. However, in this section, we have focused on the reports that mainly address stem cells.

Components of the extracellular matrix can influence the properties of stem cells, in which, for instance, the limbus promotes the development and maintenance of rLESCs (rabbit Limbal Epithelial Stem Cells). FN (Fibronectin), one component of the limbus, upregulated the expression of Ror1 and Ryk. In fact, FN acting through Wnt11/Fzd7 complex subsequently activates ROCK1 and ROCK2, improving the proliferation of rLESCs [56]. On the other hand, variations in the niche, such as oxygen levels, can affect stemness [68]. In a model of hypoxia to recapitulate the embryonic stage, Wnt5a was augmented in cardiovascular progenitor cells. Concomitantly, an increase in OCT4, SOX2, and NANOG expression was observed, suggesting a relationship between the dedifferentiated state and non-canonical Wnt pathway activation [57].

In addition to encompassing components of the extracellular matrix, the microenvironment involves cellular communication established in a bidirectional way through soluble factors secreted from the stem cells to the surrounding cells and vice-versa [58]. The upregulation of canonical Wnt signaling was found in HFSC (hair follicle stem cells) when cocultured with DPC (Dermal papilla cells). In detail, exosomes from DPCs increased the expression of β-catenin-dependent genes and augmented the proliferation and survival of HFSC, predominantly when Wnt3a was overexpressed [58]. Concerning the non-canonical Wnt pathway, HSC maintenance was partially mediated by Wnt5a secreted from urogenital ridge cells [59].

The maintenance of the stem cell pool must be ensured upon coming out of quiescence, a stage discussed in detail below, both under normal and regenerative conditions [15]. MuSC (Muscle Stem Cells) and committed myogenic precursors originate from asymmetric apical-basal cell divisions in the muscle [60]. However, it was found that Wnt7a/Fzd7 activates Vangl2 to promote stem cell expansion by symmetric division during muscle regeneration. Wnt7a did not affect TCF7 and Axin2 levels, nor the proliferation or differentiation of primary myoblasts. Interestingly, Vangl2 was absent in quiescent cells but detected along with Prickle1 and Celsr2, both interacting partners, when these cells enter the cell cycle [60]. Vangl2 had polarized localization on opposite poles of daughter cells. Still, the polarized distribution of α7-integrin suggests a mechanism through planar division by which the adherence to the basal lamina allows permanence in the niche. Meanwhile, cells oriented toward sarcolemma during apical-basal cell division move away from the niche and commit to a progenitor fate [60]. Therefore, the PCP pathway operates as a positional signal relying on the distribution of effector proteins because the absence of Vangl2 led to accelerated differentiation. Lastly, the symmetric division was unaffected by the deletion of Fzd7 in a single myofiber system. Thus, the PCP pathway could be cooperatively activated by other Wnt receptors or components such as Syn4 [60].

The role of progeny as a component of the microenvironment of stem cells is a less explored but fascinating field. Specifically, TACs (Transit Amplifying Cells) were considered a transitory state towards a differentiated lineage. Still, recently TAC was found to be an important element of the niche for stem cells, establishing that the communication between stem cells and their progeny is essential for the preservation of tissues [61]. On the one hand, canonical Wnt signaling dictates the cell fate of TAC, while on the other hand, TAC-derived Wnt5a activates non-canonical Wnt signaling through Ror2 in MSCs. In addition, MSCs regulate the timing of TACs differentiation through IGF secretion upstream of canonical Wnt signaling. Curiously, a canonical ligand Wnt10a, in addition to Wnt5a, is secreted by TACs, which could disclose an autocrine and paracrine regulation by TACs [62].

In cancer, MSC can be recruited to the tumor and exchange signals with malignant cells [83]. For example, the migratory capacity of breast cancer was stimulated upon treatment with exosomes from MSC, in which β-catenin-mediated transcription was involved [63]. On the other hand, the non-canonical Wnt pathway is relevant to convert non-malignant cells into tumor-supporting cells. LNM-GC (Lymph node metastasis-derived gastric cancer cells) reprogram BM-MSC, which are recruited to metastatic lesions, to favor lymphangiogenesis and migration of cancer cells [50]. Wnt5a-containing exosomes mediate the reprogramming by activating YAP signaling in MSC. Interestingly, high levels of Wnt5a were identified in LNM-GCs-derived exosomes and serum exosomes of gastric cancer patients with regional LNM [64]. Moreover, MSC acquires the role of chemokine sources such as CXCL12. Once CXCL12 binds to CXCR4 in cancer cells, the levels of β-catenin are augmented, triggering epithelial to mesenchymal transition [65]. According to this idea, Minami´s group reported the proliferation of undifferentiated gastric cancer cells in response to secreted CXCL16 from BM-MSC. Worthy of attention, Ror2 and Wnt5a were necessary for CXCL16 secretion [66].

MSCs are not the only non-malignant cells affected by cancer cells to promote tumor progression since macrophages can acquire a cancer cell migration-stimulating phenotype when exposed to microvesicles (MV) from patients with different cancer types, wherein Wnt5a expression occurs [67]. The phenomenon was corroborated when MV and exosomes from breast cancer cells triggered the expression of Wnt5a in macrophages. In turn, Wnt5a was transported via MV and exosomes to cancer cells, activating AP-1/c-Jun and increasing the invasiveness properties of cancer cells [68].

In regard to CSC, it has been observed that an aggressive cell subpopulation prevails upon the chemotherapeutic treatment (FOLFOX). This effect can be mediated by CAFs (Cancer-associated fibroblasts), which favor the expression of stem cell markers such as CD44V6 in CSC, by stimulating β-catenin-dependent signaling in response to Wnt3a secretion [69]. Furthermore, CAF-derived exosomes containing miR-92a-3p target FBXW7, an antagonist of β-catenin, thus upregulating the canonical Wnt pathway and eliciting stemness-related features such as the expression of CD133, CD44, and OCT4, in addition, to improve sphere-formation capacity [70]. Remarkably, the transition from somatic fibroblasts to CAFs is augmented by activation of Wnt/ β-catenin signaling caused by Wnt2B released into the exosomes of cancer cells [71]. Concerning the non-canonical Wnt pathway, macrophages induced Wnt2, Wnt5a, Fzd10, and Dvl2 expression in mammary CSC. However, the functional implication remains to be investigated [72].

**Fig. 4:**
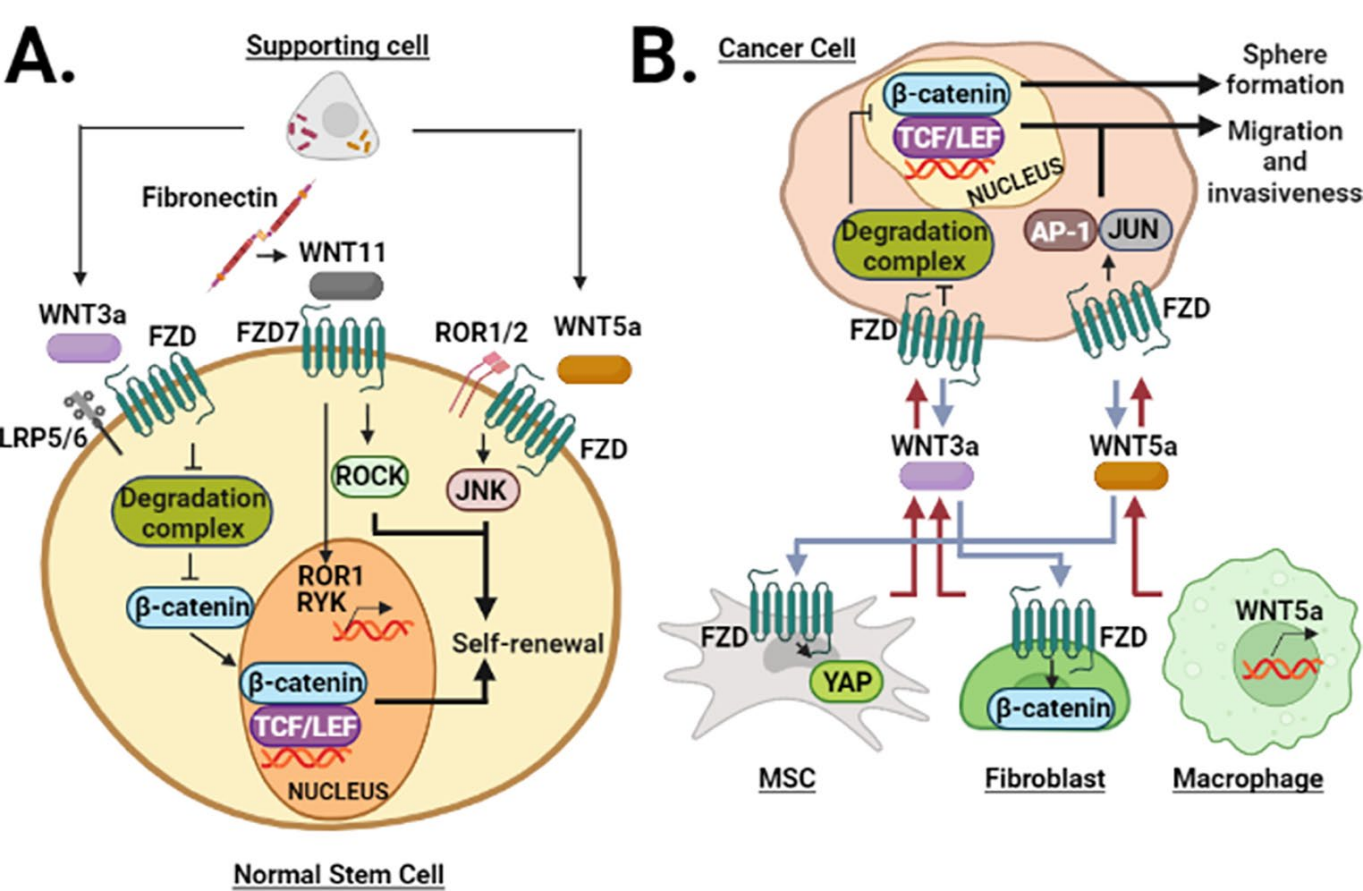
**Communication of normal and cancer stem cells with the microenvironment. (A)** Cellular components secrete Wnt3a and Wnt5a to upregulate β-catenin and JNK activities, respectively, allowing for self-renewal. Fibronectin, as an acellular component, depends on Wnt11/FZD7 complex to maintain stem cell proliferation. **(B)** Secreted Wnt3a and Wnt5a mediate paracrine communication between cancer and normal cells in the tumor, including MSC, fibroblasts, and macrophages. On the one hand, Wnt ligands transform non-malignant cells into protumoral components while stimulating migration and invasiveness in cancer cells, as well as CSC-associated capabilities, such as sphere formation

Expression of Wnt ligands is frequently analyzed in stem cells due to their autocrine action; however, the microenvironment plays a pivotal role as a source of these signals [70]. The complexity of a system where different cells interact and each has a unique expression profile of Wnt ligands has been challenging to understand under normal and pathological conditions. However, it is important to consider that the characteristics of normal and cancer stem cells are based on the communication they establish with their surroundings, both with cellular and non-cellular components, and in which non-canonical Wnt pathways functions are increasingly recognized (Fig. 4).

## The Virtue of Knowing How to Wait: Non-Canonical Wnt Pathway in the Quiescent State

Quiescence is a crucial trait of stem cells that allows them to long-term persevere and is closely related to the microenvironment [15]. Therefore, this section details the findings regarding the non-canonical Wnt pathway in quiescent stem cells. Spatial positioning is one of the critical characteristics of stem cells in relation to the niche. In the case of neural stem cells (NSC), the location is held by VCAM-1 and N-cadherin, and disruption of these molecules leads to the proliferation of NSC [73, 74]. Cdc42 activity-dependent Wnt5a maintains quiescence of NSC to express N-cadherin, thus allowing both polarity and anchoring to the apical subventricular zone [75].

The coordinated passing between proliferative and quiescent stages of stem cells must be finely balanced to meet the tissue cell turnover requirements [76]. In this regard, the hematopoietic system has allowed us to know in depth about HSCs with high and low proliferation rates, and most of the findings regarding quiescence have been obtained by studying these cells. Highly proliferating cells are the origin of differentiated hematopoietic cells, while low-proliferating cells, also known as dormant cells, replace the function of proliferative HSC when they are lost, either due to damage or disease [77]. Despite several studies exploring the relevance of the canonical Wnt pathway in hematopoiesis, the contrast of the findings reinforces the system’s complexity. Apparently, a low activation of the canonical Wnt pathway promotes self-renewal, but a high activity impairs this process and prevents terminal differentiation [77]. Quiescence in HSC has been attributed as a response to the secretion of non-canonical Wnt ligands: Wnt4, Wnt5a, Wnt5b, and Wnt11 by N-cad^+^ osteoblasts in bone marrow [78].

It was reported that a subpopulation of HSC (LSK corresponding to Lin-Sca1+Kit+ cells) expressing the non-canonical co-receptor Ryk are quiescent cells [79]. Ryk partially contributed to Wnt5a-induced quiescence and hematopoietic repopulation, and even the proportion of RYK^+^ cells diminished when HSC became multipotent progenitors. Interestingly, upon treatment with 5-fluorouracil (5- FU), an antitumor compound, Ryk^+^ HSC went into quiescence; meanwhile, RYK^−^ HSC came out. Because non-Wnt ligands for Ryk have not been described, and since the proliferation of HSC LSK by Wnt5a is disrupted, Ryk seems to be dependent on Wnt5a. Moreover, low levels of ROS were linked to Wnt5a treatment, repopulation capacity, and Ryk expression, thus emerging as an indicator of quiescence in HSC [80].

Another report found that the mode of action of Wnt5a depends on Fzd8 and Fmi to sustain the quiescent state of long-term HSC (LT-HSC). Exhaustively, Wnt5a/Fzd8 axis maintains low levels of intracellular calcium and nuclear NFAT in quiescent LT-HSC. The results denoted two possible but not mutually exclusive mechanisms: the first encompasses LTCC (L-type calcium channel), and the second involves the release of NFAT from the Cdc42/Pak1/CK1α complex [81]. Remarkably, the absence of Fzd8 stimulated the AKT-dependent phosphorylation form of β-catenin, indicating the downregulation of canonical Wnt signaling in quiescent LT-HSC. The enrichment of Fzd8 and Fmi between HSC and supportive cells was necessary to suppress nuclear NFAT, emphasizing the role of the niche in the homeostasis of HSC. Additionally, 5FU caused quiescent HSC to proliferate through the de-repression of the concomitantly canonical Wnt pathway and NFAT/calcium axis [81]. In NSC, the demyelination injury inactivates the non-canonical Wnt/Cdc42 axis to activate the canonical Wnt/β-catenin pathway, expanding the stem cell pool. This evidence corroborates the switch signaling during the transition from dormancy to reentry into the cell cycle. In the case of NSC, Wnt5a-mediated expression of Notch1 suggests it as a possible regulator of self-renewal during the quiescent state [75].

In adult muscle fibers, conditional deletion of Wnt4 demonstrated its role as a paracrine-acting niche factor, contributing to the quiescence of stem cells [47]. Interestingly, many SCs from the Wnt4-overexpressing niche remained quiescent despite exposure to mitogen stimulation. Deletion of Wnt4 in the niche did not affect canonical Wnt signaling in quiescent SCs. Nevertheless, upregulation of the phosphorylated forms of MLC (Myosin Light Chain) and FAK (Focal Adhesion Kinase), relevant to actomyosin assembly and focal adhesions, respectively, depended on Wnt4. Moreover, RhoA and ROCK contributed to maintaining the quiescence and localization of SCs within the niche. Noteworthy, the deletion of mechano-transducer YAP (Yes-Associated Protein) restored the dormancy of SC when RhoA was absent [82]. Although the function of YAP as a negative regulator of quiescence was observed, further studies are necessary because the lack of an active form of YAP has been found to improve SC proliferation [83].

**Fig. 5:**
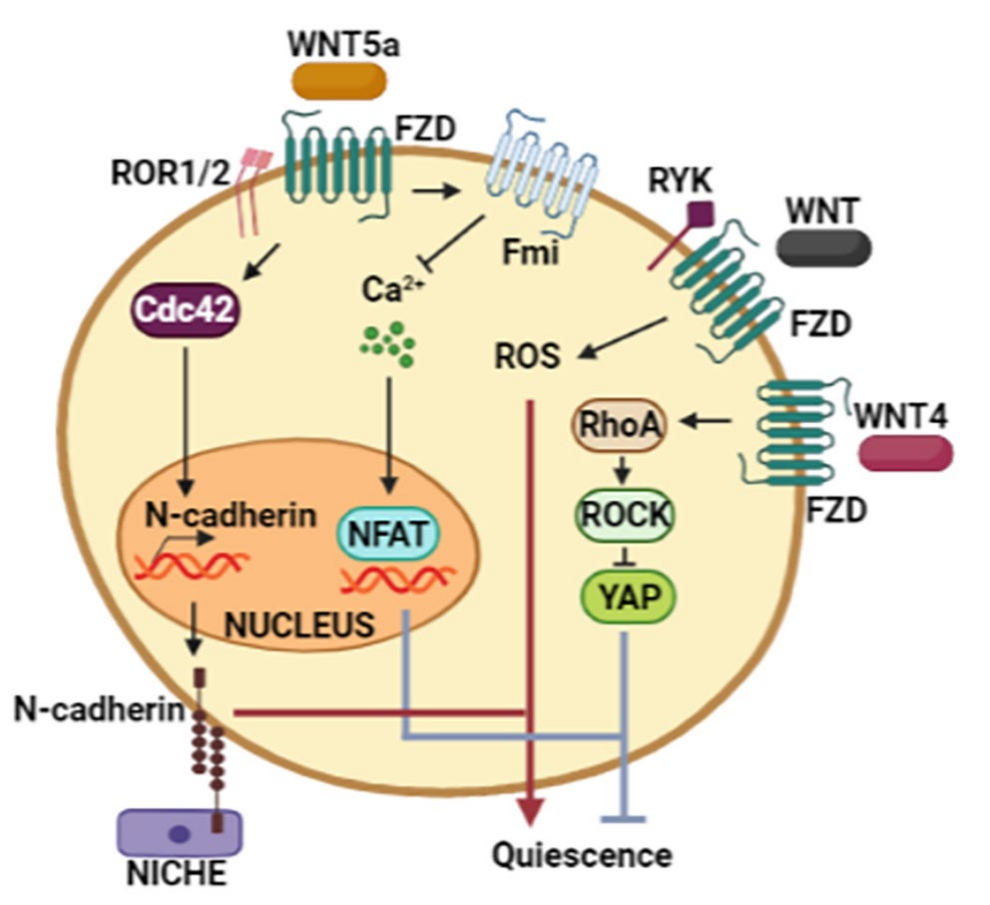
**Relationship between the quiescent state and non-canonical Wnt pathways.** Wnt5a maintains stem cells quiescence through two mechanisms. First, the upregulation of Cdc42 increases the expression of N-cadherin, which enables to keep the anchorage in the niche. Second, the reduction of intracellular Calcium-mediated by Fmi, which leads to restraining the activity of NFAT, a repressor of quiescence in HSC. Furthermore, low ROS levels are related to the quiescence state. For its part, Wnt4 activates the signaling module formed by RhoA/ROCK/YAP to augment the proliferation

There are quiescent and actively proliferating stem cells, and the transition between both states must be finely regulated by exogenous factors that dictate whether tissue conditions require stem cells to multiply. Within these factors, Wnt ligands, being communicators between cells, have been shown to participate [15]. Strikingly, the non-canonical Wnt pathways are relevant in maintaining the quiescent state, which is complicated to ascertain, because it can take up to years to remain in this way (Fig. 5).

## Time does not Stand Still: Non-Canonical Wnt Pathway as Clockwork in Senescence

The advancement of knowledge has revealed that stem cells undergo aging due to their perpetuity, which can lead to senescence. In this way, stem cell dysfunction impairs the regenerative capacity of tissues [84]. In hematopoiesis, the transition from canonical to non-canonical Wnt signaling occurs from the embryonic to the adult stage and during aging [78]. Levels of Wnt5a and Wnt4 mRNAs are higher in older than in young HSC. In aged HSC, Wnt5a was mainly localized within the cytoplasm, similar to β-catenin. Concomitantly, Cdc42 activity is triggered and associated with apolarity for Cdc42 and α-tubulin in aged HSC. In contrast, young HSC upregulated p57 and p27, markers of senescence, in response to Wnt5a, possibly by the rise of intracellular calcium and activity of CamKII and NFATc1. Meanwhile, the downregulation of Wnt5a rejuvenated aged HSC [84]. A similar phenomenon was observed in HFSC, within which Wnt5a/Cdc42 axis was activated during the aging. In aged HFSC, a reduction in the capacity of forming colonies accompanied by low levels of nuclear β-catenin and a less polar distribution of Cdc42 were described [85]. Also, the pattern of cell division is dictated by aging, being that young HSC carries it out asymmetrically, implicating differential segregation of Cdc42 into daughter cells, while aged HSC divides symmetrically. CASIN, an inhibitor of Cdc42, increased the frequency of asymmetric divisions, while Wnt5a increased symmetric divisions. Interestingly, old HSC were found in cell clusters, which could serve as niches for other HSC, while young HSC were identified as single cells. Finally, the polarity is closely related to cell division, where polar HSC undergoes asymmetric divisions and declines upon aging, while apolar HSC divides symmetrically [86].

The presence of Wnt5a as indicative of senescent cells was demonstrated in Tendon stem/progenitor cells (TSPCs). The levels of Wnt5a augmented as TSPCs enter age-related senescence [87]. In contrast, the knockdown of Wnt5a diminished senescence. In aged TSPC, the non-canonical receptor Ror2 recognizes Wnt5a, and JAK-STAT signaling acts as the mediator of the Wnt signal. For its part, β-catenin relocates from the nucleus to the cytoplasm when TSPCs age, demonstrating a switch from canonical to non-canonical Wnt signaling in these cells [87].

**Fig. 6:**
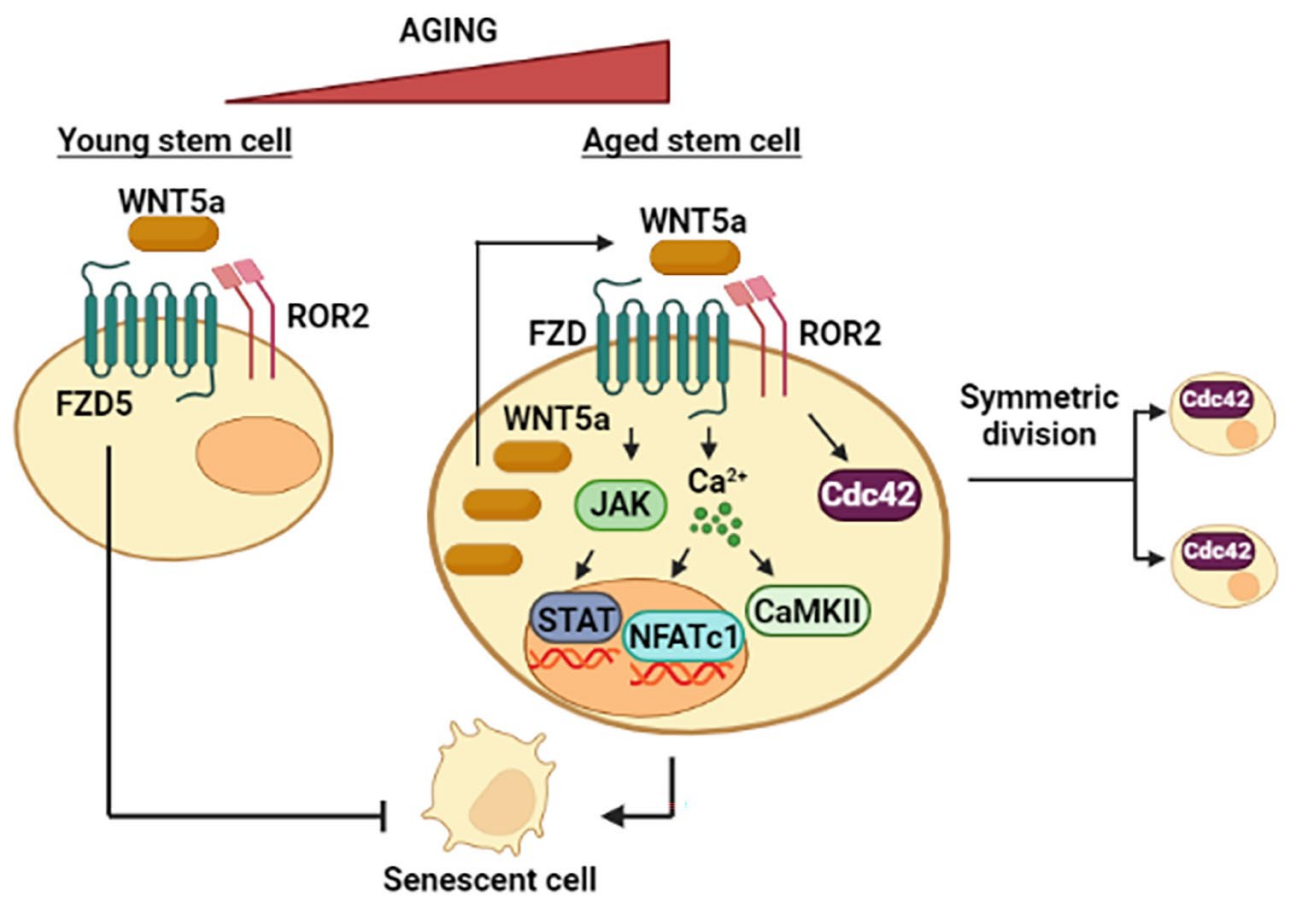
**Dual role of Wnt5a in senescence.** Wnt5a restricts entry into senescence, in which the FZD5 receptor was identified. However, high levels of intracellular Wnt5a are found in senescent cells. Specifically, JAK/STAT and Calcium/CamKII/NFATc1 are signaling axes that act downstream of Wnt5a in these cells. Cdc42, besides to respond to Wnt5a, is linked to symmetric divisions according to its distribution in the daughter cells

It is important to explain that the positive relationship between the non-canonical Wnt pathways and senescence is not yet definitive since reports are scarce. In contrast, MSC is an example of an opposite association [85–87]. The heterogeneity of MSC has allowed the identification of REC (Rapidly expanding clones), MEC (Moderately expanding clones) and SEC (Slowly expanding clones). The findings demonstrated that the Wnt5a/Fzd5/Ror2 axis was essential to maintain a high proliferation rate in REC. Additionally, senescence-related genes were upregulated, and non-canonical Wnt-associated genes, such as AP1, PLCB1, SDC1, PRKACA, and PRKCA, were downregulated upon Fzd5 knockdown. In contrast, Fzd5 overexpression inhibited senescence, demonstrating the importance of Wnt signaling for REC. Noteworthy, REC displayed c-JUN phosphorylation compared to MEC and SEC and responded to Wnt5a by activating RAC and CDC42, validating that REC is a different cell subpopulation [88]. Therefore, the phenomenon of an exacerbated activation of Wnt5a signaling that leads to senescence may not occur in each type of cell since it probably depends on the cell lineage’s characteristics, including the proliferation rate (Fig. 6).

## Stem Mask: Non-Canonical Wnt Pathway in Cancer Stem Cells

Tumors are characterized by cellular heterogeneity, within which some cells exhibit molecular and functional features of normal stem cells, like the self-renewal capacity and the ability to differentiate into specific cell lineages, but are also tumor-initiating cells. For this reason, the term CSC has been widely employed to refer to these cells [88]. Therefore, CSCs serve as the origin of the bulk of the rest of cancer cells.

Canonical Wnt pathways have been demonstrated to be important in CSCs of prostate cancer, wherein β-catenin nuclear in response to Wnt3a stimulated the size and proportion of spheres generated [89]. Furthermore, spheres of CSC obtained from glioblastoma cell lines maintain an undifferentiated state upon combined treatment with Wnt3a and Rspo2, stimulating β-catenin activity, which correlated with a higher tumorigenic capacity [90]. Similarly, Wnt3a and Rspo1 upregulated canonical Wnt target genes and stemness-related genes in hepatocellular carcinoma cells, an effect promoted by LGR5 overexpression [91]. Regarding the β-catenin-independent mechanisms, depletion of VANGL2 impaired spheres and increased differentiation markers in cells obtained from rhabdomyosarcoma [92]. VANGL2^+^ cells were enriched in the expression of FGFR3 and CD133, progenitor-related genes. The constitutively active form of RhoA reversed the effect by the absence of VANGL2. Meanwhile, activation of RAC1 had no effect, indicating that RhoA acts downstream of VANGL2 to sustain an undifferentiated state [92]. Activation of RhoA by Wnt5a serves as a survival stimulus in cancer cells during non-adherent conditions due to evasion of anoikis. In IGC (Intestinal-type gastric cancer) organoids, Wnt5a increased sphere formation, wherein Rho activity was required in the absence of Cadherin 1. Noteworthy, Cxcr4^ + ^ ILCs (Innate Lymphoid Cells) produced Wnt5a to favor the progression of DGC (Diffuse-type gastric cancer), revealing the contribution of the microenvironment [93].

As mentioned before, it was shown recently that Wnt3a and Wnt5a stimulate sphere formation in colorectal CSCs in a β-catenin-independent transcriptional activity [24]. Interestingly, the spheres-derived cells mobilized calcium in response to both Wnt ligands and pointed out Wnt/calcium signaling as an essential pathway to induce and maintain the self-renewal capacity of colon CSCs. In fact, the inhibition of phospholipase C and NFAT impaired the self-renewal in this model [24].

Another characteristic of CSC, also named TPC (Tumor-promoting stem-like cells), is the migratory phenotype to invade distant tissues, which is closely related to the aggressiveness of cancer. Xenografts of TPC obtained from glioblastoma patients exhibited invasive cells, which retained their stemness identity by maintaining the ability to form neurospheres. However, the major invasiveness was abolished by the inhibition of Wnt5a/PKC/Ca^2+^ signaling [80]. In glioblastoma, CD133 is a molecular marker for CSC. In fact, only 100 CD133^+^ cells are required to induce an invasive tumor development in vivo [94]. When comparing CD133^+^ and CD133^−^ cells, it was found that both canonical and non-canonical Wnt pathway components were expressed mainly in CD133^+^ cells, specifically RHOA, ROCK2, RAC2, and DAAM1 [95]. Accordingly, reports found impaired glioma sphere formation when RAC was inhibited [96].

Another feature related to CSC is their high capacity to efflux cytotoxic compounds, important to display chemoresistance, and frequently referred to as “side population” (SP). The percentage of SP was augmented when Wnt5a was overexpressed in nasopharyngeal cancer cells, and high rates of CD44^+^CD24^−^ cells, immunophenotype associated with CSC in some types of cancer, were detected [97]. In detail, Wnt5a increased the phosphorylation state of PKC, which in turn triggers positive feedback, upregulating Wnt5a protein levels without affecting nuclear β-catenin. In contrast, the tumorigenic potential was diminished when Wnt5a was knocked down [97].

The presence of Fzd receptors makes it possible to distinguish the involvement of Wnt pathways within the heterogeneity in tumor cells. For instance, Fzd7 expression correlated with stemness-related genes (CD44, LGR5, NOTCH2, EGFR, IL6, TNC, ANTXR1) in breast cancer [98]. Moreover, a positive correlation of Fzd7 and Wnt5b was found in cell lines and tissues of the same type of cancer. Functionally, levels of Fzd7 were directly proportional to the mammosphere formation capacity of breast cancer cell lines. Furthermore, Fzd7 interacted with Wnt5a/b, regulating the expression of surface markers such as Lgr5 and CD44, and stimulating the signaling effectors Smad3, Yap1, and Stat3. Nonetheless, it remains to be known in which processes of the CSC these effectors participate and how they regulate each other [98].

Fzd6 is another example of the relationship between Fzd family members and CSCs. A Fzd6^+^ cell subpopulation of human neuroblastoma-derived cells was identified with the potential to form neurospheres more efficiently and invasively when compared to cells lacking Fzd6 [99]. Fzd6^+^ cells expressed both canonical and non-canonical Wnt-related genes: MYC, CD44, CyclinD1, Twist, and TH (Tyrosine hydroxylase). Furthermore, FZD6^+^ cells were resistant to doxorubicin and exhibited a significant capacity for tumorigenicity and metastasis. Remarkably, the phosphorylated form of JNK was enriched in FZD6^+^ cells. Besides, the knockdown of Fzd6 decreased neurosphere formation and gene expression of CD44 and TH without affecting canonical Wnt targets MYC and Cyclin D1 [99]. It might be interesting to know the implications of Fzd6 in other cell types, such as Leukemic Stem Cells from CLL (Chronic Lymphocytic Leukemia), which also express this same receptor [100].

**Fig. 7:**
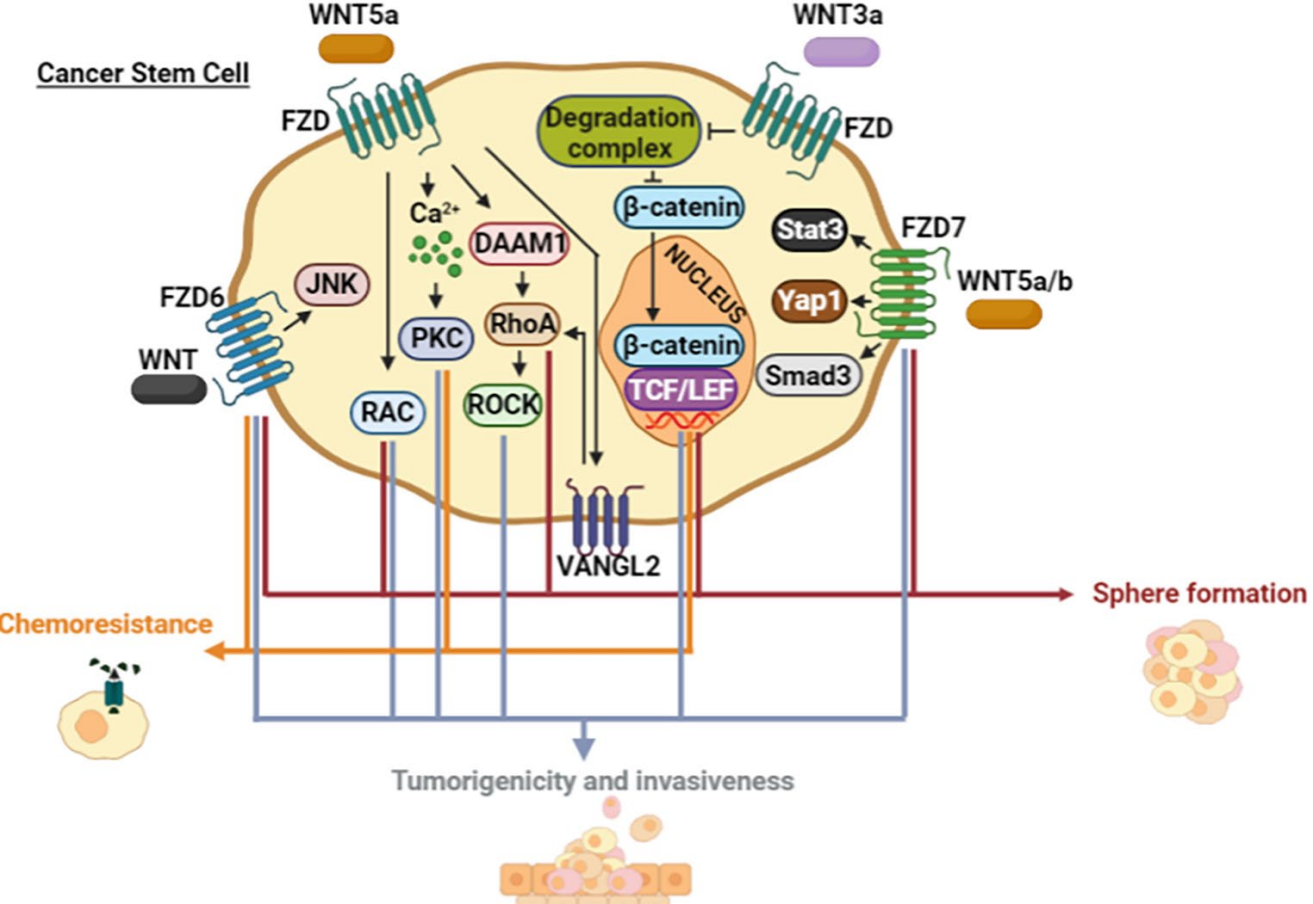
**Wnt signaling in CSC.** Wnt3a and Wnt5a improve CSC-associated features, including chemoresistance, sphere formation, invasiveness, and tumorigenicity. Non-canonical Wnt pathways encompass increased intracellular calcium and GTPases activity. VANGL2 can act as an intermediary between Wnt5a/FZD and RhoA. FZD6 and FZD7 are reported to activate β-catenin-independent mechanisms and promote CSC functionality

Exploring in-depth non-canonical Wnt pathways will allow us to know even more about altered biological processes (Fig. 7). Although cancer has been intensively explored, other health disorders are seen to be related to the functionality of these mechanisms, glimpsing its implications and possible applications in the medical field.

## Conclusions

The field of stem cell research has been expanded in recent years. During this journey, we have learned the essential roles of canonical Wnt signaling in normal and cancer stem cells. However, exploring the non-canonical Wnt pathways in stem cell biology under health and disease has taken more effort to understand its importance. Although it is well known that non-canonical Wnts regulate cell polarity, repress canonical Wnt signaling, and promote an aggressive CSC phenotype, only recently has it been demonstrated that non-canonical Wnt/calcium cascade exhibit a crucial function in inducing and maintaining the self-renewal capacity of CSCs [24].

Searching the non-canonical Wnt pathways, along with activation or inhibition of the canonical Wnt pathways, is a more comprehensive step towards understanding the global role of Wnt signaling in stem cells under conditions of health and disease. Finally, recognizing the relevance of both canonical and non-canonical Wnt signaling as a whole and as the foundation of stem-like cells in cancer could lead us to consider new strategies to fight against this disease.

## Data Availability

Within the article.
